# Unraveling the gut health puzzle: exploring the mechanisms of butyrate and the potential of High-Amylose Maize Starch Butyrate (HAMSB) in alleviating colorectal disturbances

**DOI:** 10.3389/fnut.2024.1285169

**Published:** 2024-01-18

**Authors:** Junrui Cheng, Jing Zhou

**Affiliations:** Global Scientific and Regulatory Department, Ingredion Incorporated, Bridgewater, NJ, United States

**Keywords:** resistant starch, butyrate, colorectal cancer, colitis, microbiota, gut health, digestive health

## Abstract

Colorectal disturbances encompass a variety of disorders that impact the colon and rectum, such as colitis and colon cancer. Butyrate, a short-chain fatty acid, plays a pivotal role in supporting gut health by nourishing colonocytes, promoting barrier function, modulating inflammation, and fostering a balanced microbiome. Increasing colorectal butyrate concentration may serve as a critical strategy to improve colon function and reduce the risk of colorectal disturbances. Butyrylated high-amylose maize starch (HAMSB) is an edible ingredient that efficiently delivers butyrate to the colon. HAMSB is developed by esterifying a high-amylose starch backbone with butyric anhydride. With a degree of substitution of 0.25, each hydroxy group of HAMSB is substituted by a butyryl group in every four D-glucopyranosyl units. In humans, the digestibility of HAMSB is 68% (w/w), and 60% butyrate molecules attached to the starch backbone is absorbed by the colon. One clinical trial yielded two publications, which showed that HAMSB significantly reduced rectal O^6^-methyl-guanine adducts and epithelial proliferation induced by the high protein diet. Fecal microbial profiles were assessed in three clinical trials, showing that HAMSB supplementation was consistently linked to increased abundance of *Parabacteroides distasonis*. In animal studies, HAMSB was effective in reducing the risk of diet- or AOM-induced colon cancer by reducing genetic damage, but the mechanisms differed. HAMSB functioned through affecting cecal ammonia levels by modulating colon pH in diet-induced cancer, while it ameliorated chemical-induced colon cancer through downregulating miR19b and miR92a expressions and subsequently activating the caspase-dependent apoptosis. Furthermore, animal studies showed that HAMSB improved colitis via regulating the gut immune modulation by inhibiting histone deacetylase and activating G protein-coupled receptors, but its role in bacteria-induced colon colitis requires further investigation. In conclusion, HAMSB is a food ingredient that may deliver butyrate to the colon to support colon health. Further clinical trials are warranted to validate earlier findings and determine the minimum effective dose of HAMSB.

## 1 Background

Colorectal disturbances encompass a variety of disorders that negatively impact the colon and rectum, including but not limited to colitis and colorectal cancer. An inflamed colon is a hallmark phenotype of colitis, which is a persistent gastrointestinal illness (1). Several types of colitis have been identified including ulcerative, microscopic ischemic, pseudomembranous, infectious, and neutropenic colitis, with ulcerative colitis (UC) being the most common type (2). In Europe, the annual expenses associated with ulcerative colitis, both direct and indirect, are estimated to range from €12.5 billion to €29.1 billion (3). In the United States, the estimated expenses are between US$8.1 billion and US$14.9 billion annually (3). Colitis is a risk for colorectal cancer (CRC), although the degree of association depends on disease duration and extent (4). CRC is the third most common cancer globally, and the second leading cause of cancer mortality in the United States (5). The main risk factors shared by colitis and colorectal cancer include age, being overweight or obese, a sedentary lifestyle, and unhealthy diet (6). It is well established that the consumption of a westernized diet, characterized by enriched red meat, is one of the most ubiquitous environmental factors causing UC and colorectal cancer (7).

Fibers, on the contrary, are beneficial dietary compounds that showed effects in preventing colorectal disturbances. Studies have shown that participants with a higher dietary fiber intake may have a lower risk of developing colorectal adenoma and distal colon cancers (8). Dietary fibers cannot be digested by amylase and brush border enzymes; instead, they enter the colon and be subsequently fermented by the gut microbiota. Short-chain fatty acids (SCFAs) are organic acids with fewer than six carbons, typically products of fiber fermentation. Acetate, propionate, and butyrate are the major types of SCFAs that are gaining increasing research interest. Butyrate, in particular, has attracted considerable attention as a major source of energy for colonocytes and due to its effects in modulating various health outcomes, including gut health (9), immune health (10), metabolic health (11), and cognitive and mood health (12).

Typically, starch granules are composed of amylose and amylopectin, which are two distinct types of glucose polymer. Amylose is a linear long polysaccharide consisting of α-D-glucose units that are linked through α(1 → 4) glycosidic bonds (13). Amylopectin, with a branched structure, has both α(1 → 4) and α(1 → 6) glycosidic bonds and a branch point occurring at every 25 to 30 glucose residues (13). Compared to amylopectin, amylose is less easily digested due to having fewer intramolecular hydrogen bonds for enzymes to target and a rougher surface area that blocks hydrolysis enzymes access (14). Other properties that contribute to the low-digestibility of amylose include its self-interactions during retrogradation, a native semicrystalline structure, and its capability of forming an enzyme-resistant inclusion complex with other nutrients, such as lipids, in the food matrix (15).

Derived from a special cultivar of corn, high-amylose maize starch (HAMS) contains a high portion of amylose, with levels typically ranging from 50 to 90% (16). HAMS is a type 2 resistant starch and a dietary fiber. It has been demonstrated that HAMS can escape the digestion at the small intestine and enter the colon, where it is metabolized to deliver SCFAs due to the microbial activities (17). However, In some individuals, the production of SCFAs by consuming resistant starch may be hindered as they are unable to ferment certain types of resistant starch (18). To consistently deliver the beneficial SCFAs to the colon in individuals with various fermentation challenges, chemical modification to add SCFAs to starch backbone has been shown to be an effective strategy (19). Acylated starch with specific SCFAs renders an efficient vehicle to directly deliver those SCFAs to the colon. The current work aims to review the biological characteristics of a SCFA-modified starch, butyrylated high-amylose maize starch (HAMSB), and its potentially beneficial effects in modulating colorectal disturbances.

## 2 *De novo* production, absorption, and distribution of SCFAs

**Figure 1:**
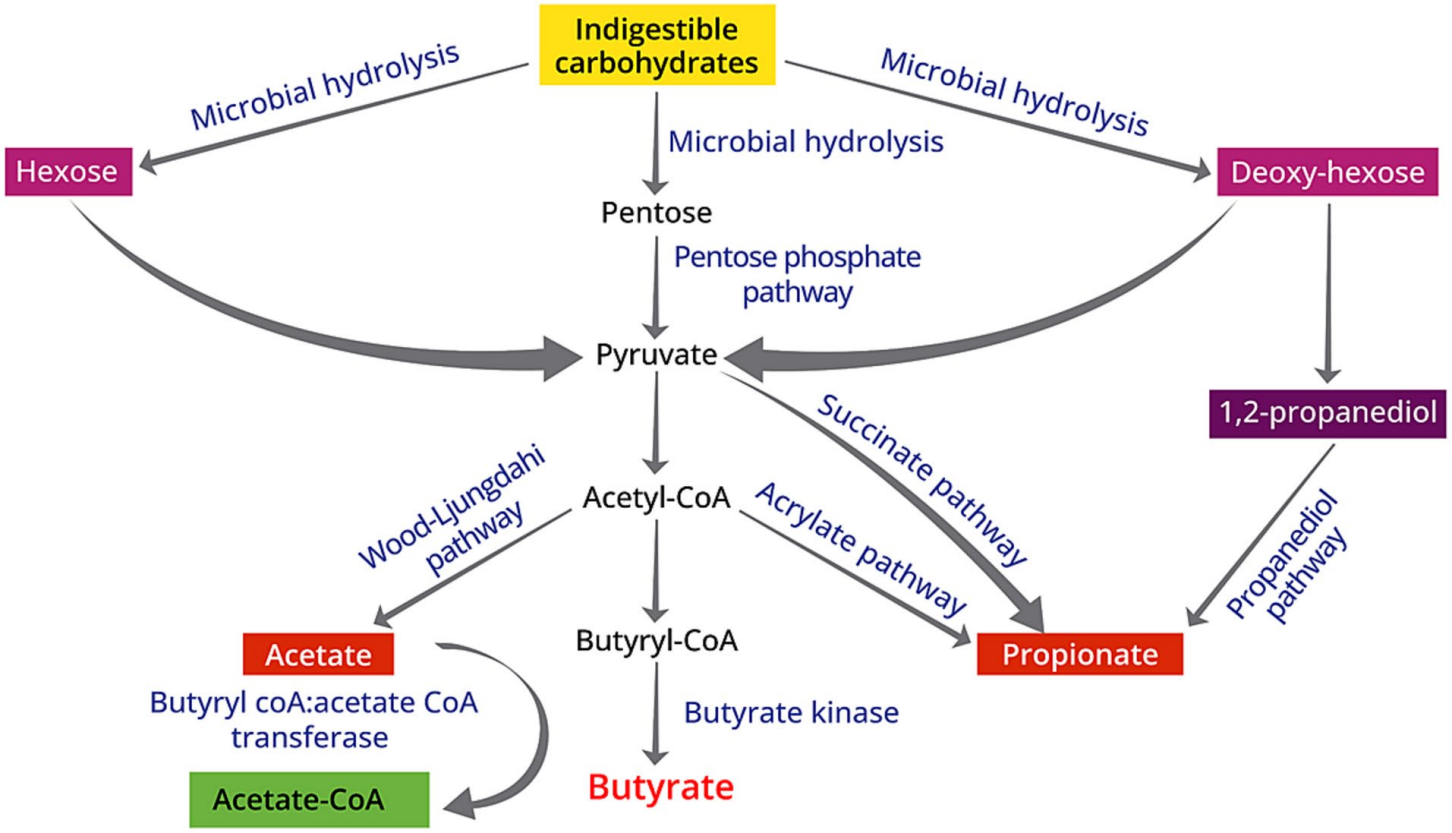
Schematic diagram of biosynthesis of SCFAs from indigestible carbohydrates due to the gut microbiota activities: (1) acetate is produced via the Wood–Ljungdahl pathway; (2) butyrate originates from the butyryl-CoA:acetate CoA-transferase pathway or the butyrate kinase pathway; (3) propionate from the acrylate pathway, succinate pathway, or propanediol pathway.

SCFAs are found in natural food sources such as ruminant milks, plant oil and animal fats (20, 21), but these volatile fatty acids are primarily produced in the gut through the anaerobic fermentation of fibers that are indigestible by the small intestine. The fermentation of amino acids also leads to the production of SCFAs, but it is accompanied with the generation of other compounds including branched-chain and aromatic amino acids, ammonia, amines, hydrogen sulfide, and phenols and indoles (22). Carbohydrate-Active enzymes (CAZymes) play a vital role in constructing and disassembling intricate carbohydrates and glycoconjugates (23), which serves as the first step of producing SCFAs. Due to their essential functions, CAZymes typically operate with a high degree of specificity, leading to different pathways of SCFAs production. This can be exemplified by the widespread presence of acetate production pathways among microbiota, compared to the limited distribution of propionate production pathways that are presented in only a few bacterial genera (24, 25). Specifically, butyrate is produced via the butyryl-CoA:acetate CoA-transferase pathway or the butyrate kinase pathway through the glycolysis of various substrates including acetate, lactate, amino acids and multiple carbohydrates (21) (Figure 1). Species such as *Akkermansia municiphilla* has been identified as a critical propionate producer, whereas *Faecalibacterium prausnitizii* and *Rominococcus bromii* are the key microbiota for butyrate production via fermenting resistant starch (25). The variation in the quantity and types of CAZyme genes expressed by different microorganisms suggests that the selective consumption of dietary fibers determines which bacterial groups are favored in the gut, affecting the balance of bacterial species and strains in the colon (26). Using equations for fermentation, the estimated daily SCFA production is about 200–600 mM based on the assumption that 20–60 g carbohydrates were fermented per day (27). Therefore, fermenting 1 g fiber may produce 10 mM SCFAs. In the United States, the average dietary fiber intake is around 16.2 g (28), indicating that the SCFA production among the United States population is at the lower end. However, it is important to note that the approximations of SCFA production in the intestine are predicated on investigations by using animal studies, which may not necessarily mirror the authentic circumstances in humans.

In the colon, where the microbial biomass is at its highest, SCFAs can accumulate to a concentration of 50–150 mM (21). Although SCFAs can be absorbed by the small intestine, colon remains to be the major site of SCFA production and absorption (29, 30). The absorption rate of SCFAs in the human rectum and descending and transverse colon is at a rate of 6.1–12.6 μmol/cm^2^ per hour (31) in a SCFA concentration-dependent manner (29). Factors that influence the absorption rate of SCFAs include the epithelial permeability to SCFAs, blood flow through the absorption surface, and the substrate composition (32, 33). A higher SCFA absorption rate is associated with increased chain length, which indicates that butyrate has the highest absorption rate among the major SCFAs (33). Approximately 60% of colonic SCFA absorption is attributed to nonionic diffusion (29), whereas the rest of SCFAs are absorbed by certain transporters in the ionized forms (21). Different SCFA transporters are selectively expressed at different segment of intestine. In the small intestine, monocarboxylate transporter (MCT)1, sodium-coupled MCT (SMCT)2, and SLC17A7 are expressed, while MCT1, SMCT2, SMCT1, and SLC26A3 are mainly expressed in the colon (34). Among these transporters, MCT1, SMCT1, and SLC26A3 have affinities for all three major SCFAs, whereas SMCT2 exclusively transports butyrate (35). The mechanisms underlying basolateral transport of SCFAs still remain unknown. The quantitative assessment suggests that the human colon exhibits the potential to assimilate a maximum of 540 kcal per day through the uptake of SCFAs (29).

The spatial variation of total SCFA concentrations in the colon was observed, showing that SCFA concentrations decrease from 70 to 140 mM in the proximal colon to 20–70 mM in the distal colon (30). The molar ratio of acetate, propionate, and butyrate is approximately 3:1:1 in the colon and stool (27, 36). The prevailing hypothesis is that almost all of the SCFAs assimilated by the colon traverse the portal vein via the colon capillaries and ultimately arrive at the liver, albeit with variable concentrations of SCFAs in the human portal vein (37). The evaluations suggest that among adults with normal liver function, the peripheral blood contains SCFAs at approximately 173 to 220 μmol/L for acetate, 4 to 7 μmol/L for propionate, and 8 to 12 μmol/L for butyrate (38). This observation indicates a significant decrease in the concentration of SCFAs in peripheral blood compared to their levels in the intestinal tract (36). The rate of SCFAs being released to the circulating system from the gut amounted to about 34.9 μmol/kg body weight per hour, which was equivalent to the rate of hepatic SCFA uptake (38), indicating that the gut and the liver are the major sites where SCFA metabolism occurs. As acetate was scarcely taken up by the liver, the molar ratio of circulating acetate, propionate, and butyrate is 9:1:1 (38), which remains stable regardless the change of body weight (38, 39).

## 3 Mechanisms by which butyrate benefits colorectal health

### 3.1 Energy source for colonocytes

Notably, the gastrointestinal milieu is principally characterized by an anaerobic milieu, affording an ecologically favorable niche for the thriving of anaerobic commensals (40, 41). Within the intricate landscape of the gut microbiome, there exists a nuanced cohabitation of both aerobic and anaerobic commensal microorganisms; however, it is noteworthy that the preponderance of the gut microbiota, constituting a staggering 99%, is comprised of anaerobic microbes (40).

It has been well-established that the gut anaerobes cannot use long-chain fatty acids for energy source. SCFAs, particularly butyrate, are important fuel for colonic epithelium (27). In the colon, butyrate can be oxidized through β-oxidation and the tricarboxylic acid cycle by the gut microbiota, partially forming ketone bodies (42, 43). Consequently, the existence of bacteria proficient in butyrate production contributes substantively to the preservation of an anaerobic milieu within the gastrointestinal tract (41), which further prevents the colonization of opportunistic aerobic pathogens, such as *Salmonella* and *E. coli* (44). This makes the colon differ from the small intestine, which does not possess the capability of oxidizing butyrate and generate ketone bodies (21).

The colonocytes have a relatively higher affinity for butyrate (43, 45), followed by ketone bodies, amino acids, and glucose, ordered from higher to lower affinity (21). Colonocytes exhibit a stronger preference for butyrate as a source of fuel in the distal colon compared to the proximal colon (43). Evidently, SCFAs impose a trophic effect on the colonic mucosa, considering that mucosal atrophy occurs after a few days of bowel rest (46). Colonocytes from patients diagnosed with ulcerative colitis exhibit a distinct defect in butyrate oxidation (47, 48). Additional investigations have reported that impaired butyrate oxidation by colonocytes could potentially induce the colorectal disturbances (49, 50).

### 3.2 Histone deacetylase inhibitor

Histone acetylation, a well-characterized approach for posttranslational histone modification, is one of the fundamental regulators of gene expression by remodeling chromatin into a state that is open and transcriptionally competent (51). This process is tightly regulated by a series of enzymes including acetyltransferases and histone deacetylases (HDACs) (52). Accumulating scientific evidence has revealed that HDAC inhibition can mitigate intestinal inflammation and inflammation-mediated carcinogenesis by suppressing the expression of proinflammatory cytokines at the site of inflammation, in conjunction with inducing specific alterations in the cellular composition of the lamina propria (53).

Apart from serving a vital source of energy for the colonocytes, butyrate possesses the capability to modulate signaling pathways through acting as an inhibitor of class I and class II HDACs (54). *In vitro* investigations showed that butyrate was found to be the most potent HDAC inhibitor among all the SCFAs (55). However, The repression of HDAC activity only impacts the expression of a small proportion, approximately 2%, of genes in mammals (56). Mechanistic investigation shows that promoters regulating genes that respond to butyrate possess specific binding sites known as butyrate response elements, the biological activity of butyrate is frequently facilitated via the interaction of Sp1/Sp3 transcription factors with these binding sites, as observed with the p21^Waf1/Cip1^ gene (56).

By inhibiting the HDAC activities, butyrate treatment affected histone decrotonylation in the intestine crypt and colon (57), and decreased malignant transformation and increased apoptosis of precancerous colonic cells (55, 58) by regulating p-21 mediated cyclin B1 expression (58). Propionate and valerate were able to induce growth arrest and differentiation in human colon carcinoma cells, but the magnitude of their effects was lower compared with butyrate (58). It has been on debate that butyrate may act as a double sword on colon health as inhibiting HDAC may affect the growth of both normal and cancerous colonocytes. However, Donohoe et al. showed that butyrate exerted opposing effects on normal cells and cancerous cells in the colon, based on their findings that the inhibition of aerobic glycolysis hindered the capability of butyrate to block normal cell proliferation, whereas the normal cells were unaffected (59). By inhibiting HDAC I, butyrate restored the activity of FoxP3 and then promoted the differentiation of naïve CD4+ T cells to maintain an optimal ratio of T helper 17 cell (Th17)/regulatory T cell (Treg) or T helper 1 cell (Th1)/Th17 (60, 61), which leads to decreased intestinal inflammation and ameliorated colon disturbances (60–62).

### 3.3 G protein-coupled receptors

Two decades ago, two orphan G protein-coupled receptors (GPR), GPR41 and GPR43, were identified as receptors for SCFAs (63). Later, it was shown that both receptors expressed in human colon epithelial cells and might mediate the SCFA-induced phasic and tonic contractions in colonic circular muscle, suggesting that the physiological effects that SCFAs impose on colon might be attributable to the activation of GPR41 and GPR43 (64). GPR109A was originally identified in an effort of exploring proteins that were differentially expressed in macrophages with different stimulations (65), but following research revealed its critical role as a receptor for butyrate, although the affinity is low (66). GPR41 has the highest affinity for propionate and butyrate, whereas GPR43 exhibits high affinity for all SCFAs, particularly propionate and acetate (63). GPR41, GPR43 and GPR109A are frequently lost in patients with colon cancer, animal cancer models, and colon cancer cells (66–68). Nevertheless, Kim et al., reported that only the knockout of GPR43, not GPR41, promoted colon carcinogenesis (69), which led the research within colorectal cancer to primarily focus on GPR43 (70).

From a mechanistic perspective, the targeting of GPR43 by propionate and butyrate resulted in a G0/G1 cell cycle arrest, accompanied by a decrease in S and G2/mitotic phases, which was achieved through the down-regulation of CDK1, CDK2, cyclin D3, and proliferating cell nuclear antigen. This process was concomitantly associated with an increase in p21, independent of p53. Additionally, propionate exhibited an ability to induce caspase 3/6/7/8 cleavage and decrease the anti-apoptotic enzyme Bcl-2. Notably, the expressions of cyclin D1, B1, 3, and CDK1 have been associated with the promotion of colon cancers (70). The activation of GPR109A signaling by butyrate has been shown to exert anti-inflammatory effects on colonic antigen-presenting cells (71, 72), which leads to the differentiation of regulatory T cells and T cells that produce IL-10, while also stimulating the production of IL-18. This subsequently alleviated colonic inflammation and colorectal cancer development (71, 72). In addition, butyrate-activated GPR109A reduced the levels of Bcl-2, Bcl-xL, and cyclin D1, while upregulating the death receptor pathway independent of HDAC inhibition. These efforts collectively promoted the apoptosis of cancer cells (66).

### 3.4 Peroxisome proliferator-activated receptor-γ

PPARs belong to a family of ligand-activated transcription factors and have three isoforms: PPAR-α, PPAR-γ, and PPAR-δ. It has been shown that butyrate treatment significantly enhanced the mRNA and protein expressions of PPAR-γ in Caco-2 cells in a dose- and time-dependent manner, which led to rapid cell differentiation (73). Similar with HT-29 cells, butyrate treatment significantly increased differentiation and inhibited cell growth by activating PPAR-γ, subsequently reduced colonic paracellular permeability and prevented colon inflammation (74). Notably, in Caco-2 cells, only butyrate treatment activated PPAR-γ; incubation with propionate and valerate did not affect PPAR-γ expression (73). However, it is currently unclear whether this selectivity is cell specific. Sodium butyrate induced autophagy both in HT-29 cells and HCT-116 cells by activating PPAR-γ, and a prolonged incubation significantly promoted cell death, particularly in HCT-116 cells (75). The variability of responses exhibited by colon cancer cells to butyrate treatment could be attributed to the dosage, incubation period, and distinctive sensitivity to differentiation of different cells that is determined by differential engagement of autophagy, caspases, and PPAR-γ signaling pathways.

In animals, the PPAR-γ signaling pathway triggered by butyrate is a homeostatic mechanism that impedes the aberrant proliferation of potentially pathogenic *Escherichia* and *Salmonella* by limiting the availability of respiratory electron acceptors to Enterobacteriaceae within the colonic lumen (76). There is a lack of research on how butyrate functions through activating PPAR- γ in humans. However, by using human colon organoids, researchers found that butyrate was capable of restoring the disrupted colonic PPAR-γ gene expression caused by hypertension (77).

In summary, butyrate is capable of manipulating the intestinal permeability, cellular growth and proliferation, as well as the gastrointestinal immune system via providing energy for colonocytes, inhibiting the HDACs, inducing the G protein-coupled receptors, and activating the PPAR-γ signaling pathways.

## 4 Butyrylated high-amylose maize starch: development and functions

### 4.1 The synthesis of HAMSB

HAMSB synthesis typically involves an organocatalytic reaction. To elaborate, a mixture of butyric acid, tartaric acid, and oven-dried corn starch is prepared at a ratio of 245:7.4:4 (w/w) and heated to 120°C in a thermostatized oil bath. Notably, tartaric acid functions as a catalyst in this process. Throughout the reaction, careful measures are implemented to ensure that distilled water washings are not initiated until the solid product has adequately cooled to prevent any potential partial gelatinization of the recovered starch esters. The degree of organocatalytic butyrylation undergoes an increase within the initial 2 h and remains at 40% acylation between 2 and 7 h. Within 2.5 h of reaction, a D.S. of 1.54 was achieved (78). Starch acetate with a DS ranging from 0.01 to 0.2 has received approval from the Food and Drug Administration (FDA) for use in food, enhancing attributes such as binding, thickening, stability, and texturizing (79). In contrast, HAMSB represents a relatively novel ingredient that has not yet secured registration with the FDA for a Generally Recognized as Safe (GRAS) status. In Australia where most studies regarding HAMSB were performed, HAMSB has not been submitted for approval for use in foods. The specific modification process determines whether it necessitates a Novel Food application with Food Standards Australia New Zealand (FSANZ). Currently, HAMSB is not registered with The Pharmaceuticals and Medical Devices Agency (PMDA) or Japan’s Specifications and Standards for Food Additives (JSFA) as a food ingredient.

### 4.2 Butyrylated high-amylose maize starch: a vehicle for butyrate delivery

**Figure 2:**
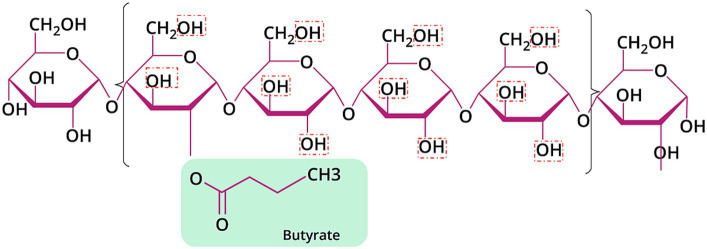
Chemical structure of HAMSB with DS of 0.25. The red dashed boxes signified the hydroxyl groups that can be substituted by butyric acid.

**Figure 3:**
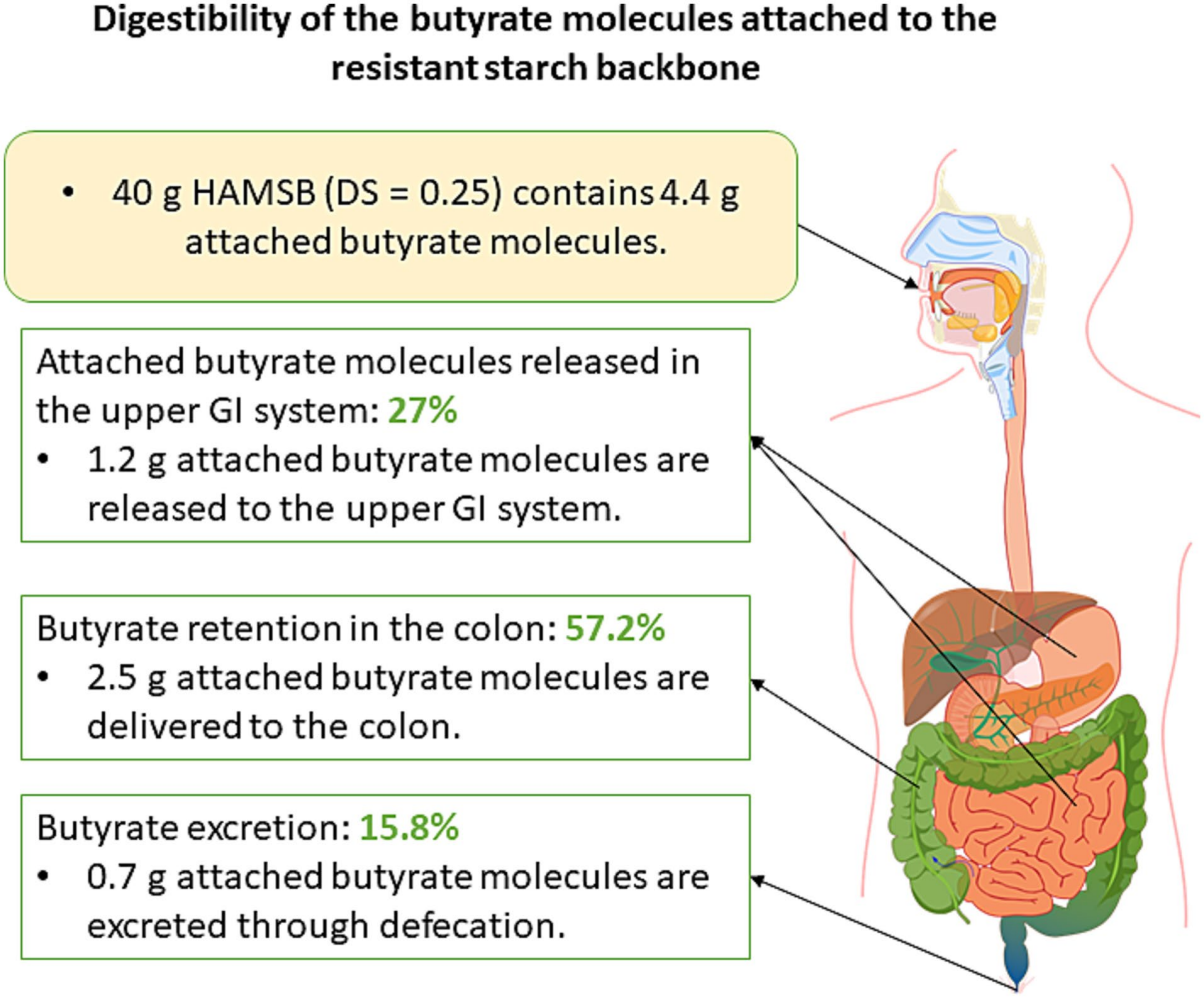
Schematic diagram that illustrates the digestibility of attached butyrate molecules. Forty-gram HAMSB was used as an example in this diagram as this was the dosage of HAMSB used in clinical trials.

The backbone of HAMSB contains about 72% amylose, which is substantially higher than the regular maize starch that typically contains 25% amylose (80). The esterification of the backbone with butyric anhydride leads to the generation of HAMSB, a SCFA-modified starch that is partly resistant to digestion in the small intestine. The degree of substitution (DS) reflects the number of hydroxy groups per each monomeric unit derivatized by a substituent (81). The DS of HAMSB is 0.25, meaning that a hydroxy group is substituted by a butyryl group in every four D-glucopyranosyl units (Figure 2). The concentration of butyrate in HAMSB is around 10% (w/w). Compared with animals fed a purified or low-amylose starch diet, animals with HAMSB supplementation exhibited significantly increased levels of acetate, propionate, and butyrate in the cecum (82–87), and a trend of increased SCFA concentrations in the distal colon (82–85). HAMS induces the production of SCFAs, but intriguingly, *in vivo* HAMSB supplementation caused a significantly higher SCFA pool in the colon (82, 85, 88–91) and circulating system (85, 88), compared with HAMS supplementation. In humans, the starch digestibility of HAMSB was around 68% (w/w), while 73% of the esterified SCFAs were indigestible in the small intestine (92), and 15.8% of was recovered in the feces when HAMSB was ingested (93). This indicates that approximately 60% butyrate molecules attached to the backbone were absorbed at the level of colon (Figure 3). However, the form of supplementation may affect the digestibility of attached butyrate molecules. For example, HAMSB released a higher amount of esterified butyrate to the colon when it was applied in milk, compared with bakery (92, 94). As SCFAs are absorbed from the human gastrointestinal tract in a concentration-dependent manner (29), increasing their concentrations within the colon through the consumption of acylated starches may yield a greater uptake compared with the consumption of comparable quantities of unacylated HAMS.

Presently, diverse delivery vehicles exist for conveying butyrate to the colon. Sodium butyrate is conventionally synthesized through an acid–base reaction, forming a salt characterized by a high melting point. Each sodium butyrate molecule yields 87 g of butyric acid. In its salt form, sodium butyrate readily dissolves in water, liberating butyrate, and ostensibly, complete butyrate release is anticipated upon dissolution. However, sodium butyrate is accompanied by an offensive odor, deemed undesirable for human consumption. Consequently, to ensure a gradual release in the intestines, sodium butyrate is commonly encapsulated within a lipid matrix coating to mitigate the unpleasant odor. Tributyrin, a precursor to butyric acid, exhibits a gradual release of butyric acid in the colon. Functioning as a triacylglyceride (TAG), tributyrin necessitates the action of lipase to release the butyrate attached to the glycerol. Despite each tributyrin molecule containing three butyrate entities, the assured release of all these moieties is not guaranteed. Lipase displays regioselectivity. While they have a degree of promiscuity irrespective of chain length and saturation/unsaturation, each enzyme can exhibit preferential or even exclusive hydrolysis of specific types of fatty acid esters (95). The reliance of tributyrin on lipase for butyrate release introduces a potential competition with other TAGs for lipase activity (96), causing the release of butyrate from tributyrin relatively inefficient. Although tributyrin is generally not coated due to its non-volatile nature at room temperature, its increased vapor pressure upon heating necessitates the use of inert silica dioxide as a carrier to preserve the intact molecule during delivery to the colon (97), concurrently masking its astringent taste. In contrast to sodium butyrate and tributyrin, High-Amylose Maize Starch Butyrate (HAMSB) represents a more natural conduit for delivering butyrate to the colon, with butyrate molecules affixed to edible starch. Furthermore, HAMSB exhibits mild odor and taste, rendering it seamlessly incorporable into various consumables such as custard, protein powder, milk, flavored milk, and orange juice without compromising flavor profiles (92–94, 98, 99). Consequently, HAMSB emerges as an advantageous candidate for butyrate delivery through integration into food and beverages.

## 5 Butyrylated high-amylose maize starch alleviates colorectal disturbances–animal and human studies

The effects of HAMSB in modulating colorectal disturbances and colon health-related biomarkers were reported by three clinical trials and 10 animal studies. Among the animal studies, eight studies focused on colon cancer and three on colitis, using various disease models (82, 84–87, 89, 90, 100–103) (Table 1). A human study explored the role of HAMSB in reducing colon cancer-related biomarkers and generated two publications (98, 99). All the clinical trials reported how HAMSB affected the gut microbial profile (93, 94, 99) (Table 2).

**Table 1:** Characteristics of the animal studies that investigated the role of HAMSB in modulating colorectal disturbances.

First author, year	Animal, animal model	Control group (backbone)	HAMSB: dosage, duration	Key findings
Bajka et al. (2006) (89)	Rat, high protein diet-induced CRC	HAMS (RS backbone)	10% diet, 10 days	↑ cecal digesta weight, ↑ cecal and distal colon acetate, propionate and butyrate concentrations. ↑ portal plasma propionate and butyrate concentrations. ↓ high protein-induced colonocyte genetic damage. ↓ cecal, proximal and distal colon pH. Affected cecal ammonia.
Clarke et al. (2008) (84)	Rat, AOM-induced CRC	LAMS; LAMS +3% tributyrin; HAMS (N/R).	10% diet, 4 weeks	HAMSB ↑ cecal tissue and digesta weight. HAMSB ↑ cecal, proximal, and distal colon butyrate; HAMSB ↑ portal plasma butyrate. HAMS, HAMSB ↓ tumor incidence compared with LAMS, HAMSB ↓ tumor number compared with LAMS. Cecal butyrate pools and concentrations were significantly and negatively correlated with the number of large bowel tumors.
Abell et al. (2011) (82)	Rat, AOM-induced CRC	HAMS (N/R)	10% diet, 31 weeks	↑ distal colon butyrate, did not change acetate, propionate concentrations. Did not change distal colon pH. Colon cancer incidence, tumor number and surface area were similar. ↑ *Lactobacillus gasseri*, *Phascolarctobacterium* and *Parabacteroides distasonis*.
Clarke et al. (2012) (85)	Rat, AOM-induced CRC	HAMS (N/R)	10% diet, 4 weeks	↑ SCFAs in large bowel digesta and plasma. ↑ apoptotic rates in the proliferate zone of distal colon (↑caspase-3), cellular proliferation did not change.
Conlon et al. (2012) (86)	Rat, Western diet-induced CRC	HAMS (Hi-Maize® 260)	28% diet, 11 weeks	↑ cecal tissue and digesta weight, ↑ cecal SCFA pool and portal vein propionate and butyrate ↓ western diet-induced weight and fat gain ↓ cecal and colon ammonia and phenols concentrations ↓ colonocyte genetic damage. ↑ *Ung*, *Gmnn*, *Cebpa* mRNA, ↓*Rere* mRNA.
Furusawa et al. (2013) (100)	Mouse, genetic modification-induced colitis	HAMS (N/R)	15% diet, 4 weeks	↓ colitis Induced Treg cells independent of TLR-MyD88 pathway ↑ histone H3 acetylation in the promoter and conserved non-coding sequence regions of the Foxp3 locus.
Toden et al. (2014) (87)	Rat, AOM-induced colon cancer	LAMS (AIN-93G)	5, 10, 20, 40%, 4 weeks	↑ Gut total SCFA, acetate and butyrate pools; ↑ hepatic portal venous plasma total SCFA, acetate, butyrate pools, ↓cecal ammonia pools. ↑ distal colonic epithelial apoptotic index, mucus thickness. ↓ Genetic damage dose-dependently; ↑ apoptotic rates, not affect colonocyte proliferation.
Le Leu et al. (2016) (102)	Rat, AOM-induced CRC	LAMS (AIN-93G)	20% diet, 4 weeks	↓ AOM-induced O_6_MeG adducts, especially in the lower third of the crypts. Crypt column height did not change. ↑ apoptotic rates
Nielsen et al. (2019) (99)	Rat, high protein diet-induced CRC	HAMS (Hi-Maize® 260)	10% diet, 4 weeks	↓ cecal acetate, not affect propionate, ↑ cecal butyrate, ↓ branched-chain fatty acids, ↑ fecal output. ↓ Diversity, ↑ Proteobacteria Sutterella, Proteobacteria Bilophila, Parabacteroides. ↓ miR19b and miR92a, ↓ O_6_MeG formation (not statistically significant).
Isobe et al. (2019) (101)	Mouse, DSS-induced colitis	HAMS (N/R)	15% diet, 4 weeks	↓ the translocation of luminal bacteria to the liver. ↑ IgA production in the colonic lamina propria by ↑ the T-cell independent response, which was mediated by GPR41 and GRP109a/HCA2, and the inhibition of HDAC. ↑ colonic barrier function; ↓ systemic bacterial dissemination under inflammatory conditions.
Yap et al. (2021) (103)	Mouse, *Citrobacter rodentium* infection- induced colitis	HAMS (N/R)	15% diet, 3 weeks	Did not change infection-induced weight loss. ↑ epithelial damage of distal colon, ↓ neutrophils at lamina propria.

AOM, Azoxymethane; DSS, dextran sulfate sodium; GPR: G protein-coupled receptor; HAMS, high-amylose maize starch; HAMSB, Butyrylated high-amylose maize starch; HDAC, histone deacetylase; O_6_MeG, O_6_-methyl guanine; N/R, not reported; SCFA: short-chain fatty acid.

**Table 2:** Characteristics of the human clinical studies that investigated the role of HAMSB in colorectal disturbances.

First author, year	Number of subjects	Dietary groups and dosages	Duration	Key findings
Clarke et al. (2011) (94)	16	-Control: HAMS 20 g/d or 40 g/d; -Intervention: HAMSB 20 g/d or 40 g/d.	2 weeks^a^	1. Free and esterified butyrate concentrations were highest in HAMSB40, and were overall higher in the HAMSB groups. 2. ~57.2% of ingested esterified butyrate was released in the colon when the subjects consumed HAMSB at 40 g/d. 3. ↑ *Parabacteroides distasonis* at both dosages.
West et al. (2013) (93)	23	-Control: Low-amylose starch, 40 g/d; -Intervention: HAMSB, 40 g/d.	4 weeks	1. Saliva IgA, lysozyme, lactoferrin did not change. 2. ↑ plasma IL-10 and TNFα, − IL-1RA, IL-6, IL-8, or granulocyte macrophage-colony-stimulating factor (GM-CSF). 3. ↑ *Parabacteroides distasonis*, *Faecalibacterium prausnitzii* 4. ↑ Fecal output, fecal acetate did not change, ↑ fecal propionate, free/bound/total butyrate.
Humphreys et al. (2014) (98)	23	-Control: HRM, 300 g/d; -Intervention: HRM, 300 g/d + HAMSB, 40 g/d	4 weeks^a^	1. ↑ fecal SCFAs (acetate, butyrate, propionate) 2. ↓ miR 17, miR19a, miR20a, miR19b, miR92a; 3. ↓ cdkn1a, ↑ pten, bcl2l11 mRNA levels, PCNA (all NS) 4. A significant effect of treatment order: HRM + HAMSB first group had significantly less proliferation compared with the HRM first group.
Le Leu et al. (2015) (99)	23	-Control: H, 300 g/d; -Intervention: HRM, 300 g/d + HAMSB, 40 g/d	4 weeks^a^	1. ↓ HRM-induced rectal O^6^MeG adducts and epithelial proliferation; 2. ↑ total fecal SCFA, acetate, butylrate, propionate, and ammonia excretion, − N-nitroso compounds; 3. ↑ *Parabacteroides distasonis* and *Ruminococcus bromii*, ↓ *Ruminococcus torques*, *Ruminococcus gnavus*, and *Escherichia coli*.

^a^Cross-over study. HAMS: high-amylose maize starch; HAMSB: Butyrylated high-amylose maize starch; HRM, high red-meat diet; NS: non-significant.

### 5.1 Butyrylated high-amylose maize starch changes microbial composition

Animal and human studies that examined the effects of HAMSB in modulating the gut microbial composition consistently reported a significantly increased relative abundance of *Parabacteroides distasonis* in the HAMSB-supplemented group, compared with that without HAMSB supplementation (82, 90, 93, 94, 99). Interestingly, the treatment of acetylated-HAMS (HAMSA) or a combination of HAMSA and HAMSB also showed an increased abundance of *P. distasonis* (103, 104). Nevertheless, this species was not selectively improved by supplementing butyrate alone (105), suggesting that the starch backbone might play a role. The bacterial strain *P. distasonis* serves as the reference organism for the taxonomic category of Parabacteroides, a class of anaerobic, gram-negative bacteria that are frequently present in the gastrointestinal tracts of various species (106). Recent studies showed that *P. distasonis* were lower in patients with certain diseases, including multiple sclerosis (107) and colorectal cancer (108), but the causality remains unknown. There have been reports indicating that *P. distasonis* may exhibit probiotic properties capable of promoting digestive health in humans, as demonstrated by *in vitro* and *in vivo* studies (106). Nonetheless, divergent experimental data have also been presented, which suggest the potential for pathogenic effects in diverse disease models. Such observations indicate that *P. distasonis* may exhibit a dichotomous role contingent upon the context of its interaction with the host, including factors such as the host’s susceptibility to immune suppression and impaired bacterial clearance, as well as the promotion of hyperinflammatory responses. Additionally, strain-to-strain variations may play a role in accounting for potential differences in its pathogenicity (106).

Among humans with HAMSB supplementation, other commensal bacteria including *Faecalibacterium prausnitzii* (93) and *Ruminococcus bromii* (99) were found increased, while certain bacterial species including *Ruminococcus torques*, *Ruminococcus gnavus*, and *Escherichia coli* were reduced (99), but the results were inconsistent. *F. prausnitzii* has been consistently identified as a principal butyrate producer (109) and shown to mitigate the severity of inflammation by producing metabolites that enhance the mucosal barrier function and decrease the intestinal permeability (110). *R. bromii* is a pivotal species that plays a crucial role in the process of breaking down resistant starch within the human colon (111). The increased *F. prausnitzii* and *R. bromii* may be attributed to the consumption of the backbone itself. In animals, HAMSB treatment significantly enhanced genus *Bacteroides* (91, 112–114) and *Blautia* (91, 113). However, caution is warranted for data interpretation as the animal studies used heterogenous disease models.

### 5.2 Butyrylated high-amylose maize starch reduces the risks for colorectal cancer

High consumption of red meat (115) and western dietary patterns (116) are associated with increased risks of CRC. The occurrence of the O^6^-methyldeoxyguanosine (O^6^-MedG) lesion, which is recognized as an indicator of exposure to numerous N-nitroso compounds, is frequently detected in tumor DNA isolated from colon tissue (117). Two publications generated by one study showed that HAMSB significantly reduced rectal O^6^-MedG and epithelial proliferation induced by the high red meat diet (300 g lean beef per day), potentially by inhibiting microRNA (miR) 17, 19a, 20a, 10b, and 92a, and modulate the genes in cell cycle control. Notably, rectal miR17-92 cluster miRNAs have been found elevated in CRC (118, 119) and are linked with invasion and metastasis of colon cancer cells (120) and a higher risk of cancer-related death (119). Using diet-induced CRC models, researchers consistently reported beneficial effects of HAMSB supplementation in alleviating colonocyte DNA damage (86, 89, 90) and reducing O^6^-MedG formation, which were associated with decreased miR19b and 92a (90) that might be modulated by histone hyperacetylation (121). However, it needs to be mentioned that in the United States, the total red meat consumption is around 0.74 servings per day in women and 1.03 servings per day in men (122), a dosage that is substantially lower than the amount of red meat given to the subjects in the trials. Therefore, in future studies investigating the relationship between diet and the development of colorectal cancer, it is advisable to utilize a reduced amount of red meat to better reflect its impact on public health.

Azoxymethane (AOM) is the most commonly utilized carcinogen to simulate the progression of sporadic CRC (123), which represents the 90–95% of CRC cases (124). HAMSB was found to be effective in reducing AOM-induced CRC risk in four animal studies (82, 84, 85, 87, 102), where elevated apoptotic rates were consistently observed (85, 87, 102) with a higher caspase-3 expression (85). Caspases are fundamental regulators of programmed cell death, with caspase-3 being a frequently activated death protease that facilitates the targeted cleavage of numerous essential cellular proteins (125), and can be induced by histone deacetylase inhibitors including butyrate (126). Therefore, it is possible that HAMSB, acting as a HDAC inhibitor, mitigated AOM-induced colon carcinogenesis by promoting caspase-3 associated apoptosis. Intriguingly, while HAMSB showed anti-CRC effects in animals, tributyrin exhibited no impact on colon tumor development (84). Notably, at the concentration of tributyrin included in the LAMS diet in this study (3%), hepatic portal plasma butyrate concentrations were comparable to those achieved through the ingestion of the HAMS diet and were than those achieved through the consumption of the HAMSB diet. The data suggest that HAMSB could be a more efficient carrier for delivering butyrate compared to tributyrin.

Most studies that quantified colon metabolites reported a reduced level of cecal ammonia in the animals supplemented with HAMSB (86, 90). Ammonia is recognized as a carcinogenic agent that can induce colon mucosal cell damage (127, 128) by improving the colonic pH (129). HAMSB treatment led to a lower cecal and distal pH (88, 89), which may contribute to eliminating ammonia and preventing colonic carcinogenesis.

### 5.3 Butyrylated high-amylose maize starch and colon colitis

The role of HAMSB in modulating colitis was examined by three studies using different animal models. Researchers found that HAMSB was beneficial in mitigating genetic modification induced colitis (100) and dextran sulfate sodium (DSS)-induced colitis (101) through activating innate and adaptive immune responses (100, 101). In specific, HAMSB favored the differentiation of naïve T cells into regulatory T (Treg) cells through the stimulation of histone H3 acetylation within both the promoter and conserved non-coding sequence regions of the Foxp3 locus in the Rag1 knockout mice that received the adaptive transfer of CD4^+^CD45RB^hi^ T cells (naïve T cells) (100). In the mice injected with DSS, HAMSB intake significantly promoted IgA production in the colonic lamina propria by conditioning dendritic cells and intestinal epithelial cells (101). This effect was mediated by GPR41 and GPR109a activation as well as epigenetic modification (101).

However, in the study conducted by Yap et al., HAMSB did not ameliorate colitis induced by *Citrobacter rodentium* infection (103). *C. rodentium* is a Gram-negative species of bacteria in rodents that shares several pathogenic mechanisms with *E. coli*, making it a valid model to investigate common human intestinal diseases (130). However, the finding needs to be validated with more studies as this result was in contradiction with the *in vitro* data where butyrate significantly inhibited the growth of *C. rodentium* in a dose-dependent manner (103).

### 5.4 Butyrylated high-amylose maize starch improves mucosal barrier

Mucosal barrier is a semipermeable structure that functions through the combined effects of multiple extracellular and cellular processes to establish physical and chemical defenses against toxins and pathogens. In the context of an intact epithelium, tight junction barrier function represents the principal factor governing mucosal permeability (131).

In mice with DSS-induced colitis, HAMSB supplementation substantially enhanced colonic barrier function and inhibited the translocation of luminal bacteria to the liver by reducing systemic bacterial dissemination (101). Feeding the depressed mice with HAMSB that was produced by utilizing HAMS as backbone, Tian et al. reported elevated mRNA levels of *claudin* and *occludin* (114), which are crucial tight junction proteins that regulate intestinal permeability. In a model of type I diabetes, dietary HAMSB significantly enhanced the colonic *occludin* mRNA expression and decreased lipoprotein saccharide concentration in the peripheral blood (112). Although these studies shed light on the mechanism by which HAMSB improved colon health, they only detected the biomarkers of the mucosal barrier; the dual sugar absorption test should be employed as the gold standard test for intestinal permeability to validate the effects of HAMSB in modulating the epithelial barrier function.

**Figure 4:**
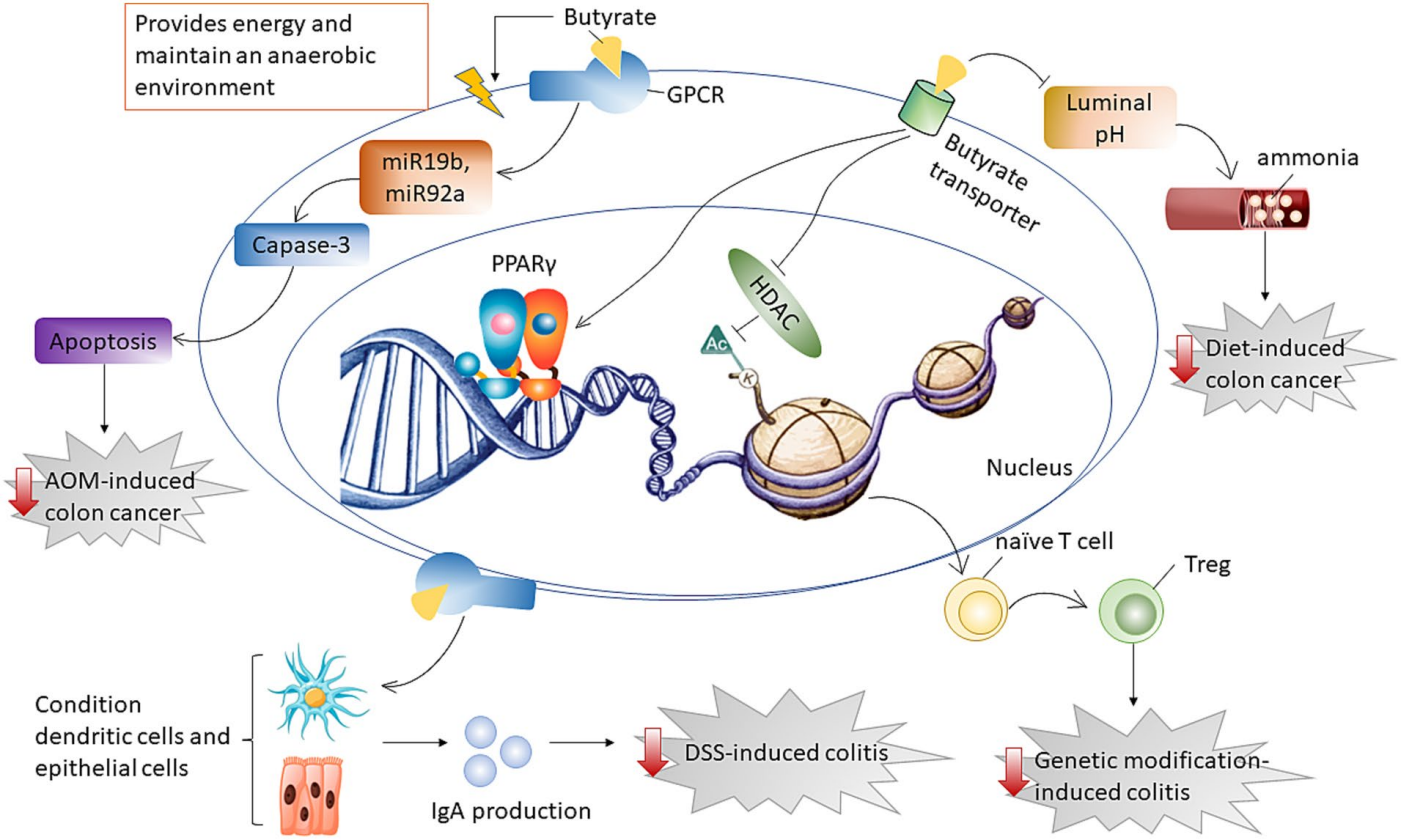
Diagram that illustrates the mechanism by which butyrate supports gut health. Butyrate delivered by HAMSB was effective in alleviating diet-induced cancer by affecting cecal ammonia levels whereas ameliorating AOM-induced colon cancer by inducing cancer apoptosis through downregulating miR19b and miR92a. Butyrate mitigated genetic modification-induced colitis by inhibiting HDAC for epigenetic modification, while alleviated DSS-induced colitis through improving IgA release that was generated from conditioning dendritic and epithelial cells.

Overall, HAMSB was effective in reducing the risk of diet- or AOM-induced colon cancer through different mechanisms. HAMSB alleviated diet-induced cancer by affecting cecal ammonia levels whereas ameliorated AOM-induced colon cancer by inducing cancer apoptosis through downregulating miR19b and miR92a. HAMSB mitigated genetic modification-induced colitis by playing a role as HDAC inhibitor, while alleviated DSS-induced colitis through conditioning dendritic cells and epithelial cells and subsequently improving IgA release (Figure 4).

## 6 Discussion and future direction

The current work reviews the function of HAMSB, an edible ingredient that efficiently delivers butyrate to the colon. We also reviewed the research that examined the role of HAMSB in supporting colon health. Together these studies paint a positive picture for HAMSB in alleviating colorectal disturbances including CRC, colitis, and gut dysbiosis. Further studies are warranted to validate the function of HAMSB in modulating pathogenic bacteria infection-induced colon diseases.

In humans, approximately two-thirds of the HAMSB were digested in the small intestine (92), which was higher than what was reported in an animal *study*, where half raw acylated starches escaped the digestion in the upper GI tract of the colectomized rats (132). The discrepancy might be due to the high temperature during cooking, as it was reported that cooking decreased the indigestibility of HAMS in the small intestine from 64 to 28% (133). Importantly, the digestibility of starchy foods is influenced by multiple factors such as food matrix, moisture, storage conditions, and processing methods (134). Hence, it would be valuable to investigate the impact of cooking methods on the structure and digestibility of HAMSB with more studies to understand its application in food and beverages.

**Figure 5:**
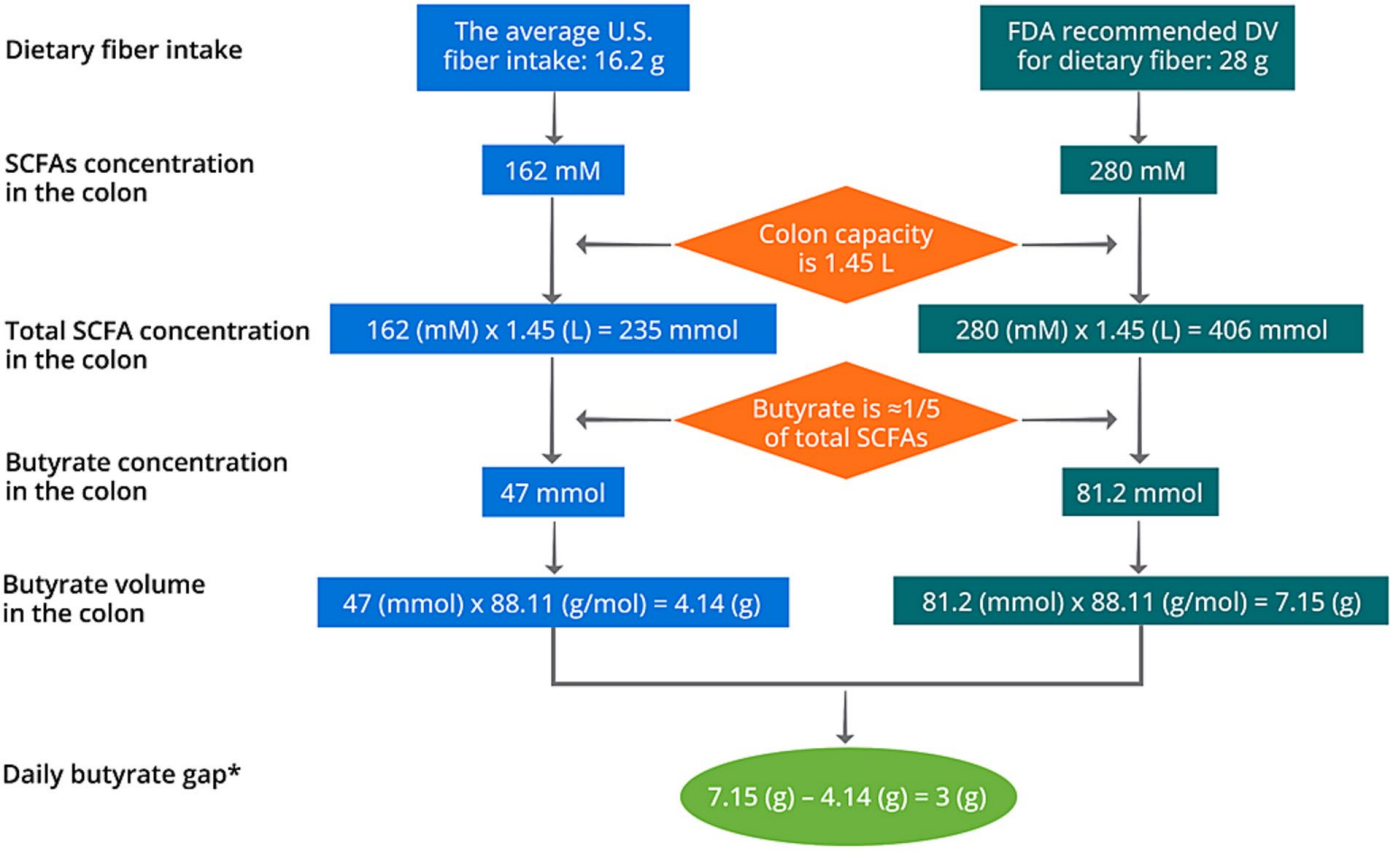
Schematic diagram of the calculation of daily butyrate gap in the United States. *The daily butyrate gap solely represents the short of butyrate production based on fiber deficit. It does not indicate that the health outcomes generated from fiber deficit is a result from the gap of colorectal butyrate production.

Although colon can absorb SCFAs at a rapid rate and high amount (30, 31), around 15% butyrate were excreted with a supplementation at 4 g/d (93). This indicates that a lower dose of butyrate supplementation at around 3.4 g/d might be optimal. Notably, individual variance may exist in the capability of absorbing SCFAs, as recent studies have identified polymorphisms in several SCFA transporters including MCT1 and MCT2 genes (135, 136). As mentioned in Section 2, the fermentation of 1 g fiber may correspond to the production of 10 mM SCFAs. Therefore, consuming 16.2 g dietary fiber may generate 162 mM SCFAs in the colon. By assuming a colon capacity of 1.45 L [1.4 L for healthy female and 1.5 L for healthy male (137)], the daily butyrate production is around 4.14 g based on the average United States fiber intake of 16.2 g/d (28) (Figure 5). In the United States, the daily value (DV) of dietary fiber is designated at 28 g, meaning that the United States population is recommended to consume at least 28 g/d dietary fiber on most days. Such fiber deficit may result in a gap of butyrate production of 3 g/d (Figure 5). Typical butyrate supplements in the market deliver butyrate at a daily dosage of 150–300 mg, which may not cover the demand and an increase in dosage of supplementation should be considered (138), preferably at 3–3.4 g/d based on our calculation. Nevertheless, this does not indicate that any changes of health outcomes resulted from fiber deficit is causally associated with colorectal butyrate production. Future prospective cohort studies and clinical trials are warranted to identify the causal relationship between the butyrate deficit, the dosage gap, and potential negative health outcomes.

There has been debates about whether the circulating SCFAs or colon SCFAs confer greater health benefits. Acetate, propionate, and butyrate exhibit considerable agonistic activity on G protein-coupled receptors and PPAR-γ when compared to other SCFAs (i.e., branched SCFAs), with an EC_50_ of around 0.5 mM (63, 139–141). The activation of these receptors necessitates millimolar concentrations of SCFAs, indicating a low potency in comparison to other G protein-coupled receptor ligands such as the CCL chemokine, which activates the chemokine receptor CCR2 with an EC_50_ of around 1 nM (142). As a result, the activation of GPR41, GPR43, and PPAR-γ may be limited to specific areas within the human body (143), such as in the gut lumen where SCFA concentrations are greater than 20 mM (21, 30). As the most potent HDAC inhibitor, the IC_50_ of butyrate is around 30–90 μM (55, 144), a concentration that is unachievable in the circulating system even with HAMSB supplementation that conferred butyrate at a dosage of 4 g per day (145). Therefore, it suggests that the colon is the primary site where SCFAs perform physiological, biological, and immunological modulations. Delivering the SCFAs to the colon efficiently is critical to enable SCFAs to function properly.

The studies list in the present work have several limitations. First, two animal studies used Hi-Maize® 260 instead of the resistant starch backbone as positive control (86, 90). Hi-Maize® 260 is physically modified by using the resistant starch backbone as a base starch (146). Compared with this starch backbone, Hi-Maize® 260 has a comparable concentration of amylose but an increased level of RS, which may elicit a more potent apoptotic response to AOM in the colon of rats (146). Thus, using Hi-Maize® 260 as control may decrease the effect size and increase the possibilities of observing null results. This suggests that HAMSB might have more compelling effects in alleviating colorectal disturbances than what were reported by the existing studies. Another limitation stems from the fact that HAMSB supplementation enhanced other SCFAs including acetate and propionate in the colon and circulating system (86, 89), which renders challenges to investigate the health benefits that are incurred by butyrate alone. However, such limitation does not defeat the conclusion that HAMSB as an intact dietary compound can improve colon health.

In addition, all the clinical trials that evaluated health-related endpoints used HAMSB at a dosage ≥40 g/d, which requires the subjects to take multiple servings of food to reach the designated amount (92). The animal studies provided HAMSB at a range of 10–28% diet, which is equivalent to 181–507 g/d HAMSB (18.1–50.7 g/d butyrate) in humans by assuming that four pounds of food is consumed each day. Since the physiological range of oral butyrate supplementation is 1–10 g/d (138), these animal studies lack physiological relevance. Future research should focus on exploring the minimum effective dose of HAMSB or its dose–response effects. It’s important to note that the number of studies investigating the effectiveness of HAMSB in alleviating colorectal disturbances is limited, and the majority of these studies are conducted on animals, which generated a logical leap generated from extrapolating the results from animal studies to humans. Rodents exhibit a larger body surface area and weight relative to humans, thereby manifesting an augmented metabolic capacity. In toxicology studies, administration of dosages denoted as “human equivalent doses” is a customary practice. Specifically, these doses are calibrated to be 12.3 and 6.2 times the equivalent human dose when administered to mice and rats, respectively (147). While murine have adapted to an enlarged colon and cecum capacity, allowing them to extract additional nutrients from a comparatively higher proportion of indigestible food components in their diet compared to humans (148), they may exhibit intolerance to components flowing excessively intact from the small intestine into the colon. Thus, the appropriateness of such dosages for animals is contingent only when the test component is absorbed in the small intestine, and are ineffective when the components’ functionality is dependent on the intestinal bacteria within the hosts. Consequently, it would be premature to consider HAMSB as a standalone solution for addressing colorectal disturbances. Instead, the main emphasis should be on adopting a healthier diet and lifestyle. Further clinical trials are necessary to establish and validate the potential effects of HAMSB in promoting colon health.

In conclusion, HAMSB is an edible ingredient that can efficiently deliver butyrate to the colon. Existing clinical trials and animal studies suggest that HAMSB supplementation at a dosage equal or larger than 40 g/d may mitigate dysbiosis, fortify mucosal barrier, and reduce the risks for colorectal cancer and colitis. Therefore, it serves as a promising dietary strategy to support gut health. Future studies are warranted to validate such findings with additional clinical trials and a lower dosage of HAMSB.

## Author contributions

JC: Writing – original draft, Writing – review & editing. JZ: Writing – review & editing.
